# White matter change with apathy and impulsivity in frontotemporal lobar degeneration syndromes

**DOI:** 10.1212/WNL.0000000000005175

**Published:** 2018-03-20

**Authors:** Claire J. Lansdall, Ian T.S. Coyle-Gilchrist, P. Simon Jones, Patricia Vázquez Rodríguez, Alicia Wilcox, Eileen Wehmann, Katrina M. Dick, Trevor W. Robbins, James B. Rowe

**Affiliations:** From the Departments of Clinical Neurosciences (C.J.L., I.T.S.C.-G., P.S.J., P.V.R., A.W., E.W., J.B.R.) and Psychology (T.W.R.), and Behavioral and Clinical Neuroscience Institute (T.W.R., J.B.R.), University of Cambridge, UK; University Medical Centre Hamburg-Eppendorf (E.W.), University of Hamburg, Germany; The Dementia Research Centre (K.M.D.), Institute of Neurology, University College London; and MRC Cognition and Brain Sciences Unit (J.B.R.), Cambridge, UK.

## Abstract

**Objective:**

To identify the white matter correlates of apathy and impulsivity in the major syndromes associated with frontotemporal lobar degeneration, using diffusion-weighted imaging and data from the PiPPIN (Pick's Disease and Progressive Supranuclear Palsy: Prevalence and Incidence) study. We included behavioral and language variants of frontotemporal dementia, corticobasal syndrome, and progressive supranuclear palsy.

**Methods:**

Seventy patients and 30 controls underwent diffusion tensor imaging at 3-tesla after detailed assessment of apathy and impulsivity. We used tract-based spatial statistics of fractional anisotropy and mean diffusivity, correlating with 8 orthogonal dimensions of apathy and impulsivity derived from a principal component analysis of neuropsychological, behavioral, and questionnaire measures.

**Results:**

Three components were associated with significant white matter tract abnormalities. Carer-rated change in everyday skills, self-care, and motivation correlated with widespread changes in dorsal frontoparietal and corticospinal tracts, while carer observations of impulsive–apathetic and challenging behaviors revealed disruption in ventral frontotemporal tracts. Objective neuropsychological tests of cognitive control, reflection impulsivity, and reward responsiveness were associated with focal changes in the right frontal lobe and presupplementary motor area. These changes were observed across clinical diagnostic groups, and were not restricted to the disorders for which diagnostic criteria include apathy and impulsivity.

**Conclusion:**

The current study provides evidence of distinct structural network changes in white matter associated with different neurobehavioral components of apathy and impulsivity across the diverse spectrum of syndromes and pathologies associated with frontotemporal lobar degeneration.

Apathy and impulsivity are common and often coexistent in neurodegenerative disorders, including the clinical syndromes resulting from frontotemporal lobar degeneration (FTLD).^1–3^ They are difficult to treat and cause substantial patient morbidity and carer distress.^4^ Research into the causes and treatment of apathy and impulsivity is challenging because they are both multifaceted constructs: apathy reflects abnormal goal-directed behavior, from dysfunction in cognitive, emotional, and behavioral domains,^5^ while impulsivity is the tendency to act prematurely, without forethought or appropriate consideration of risk.^6^

Resolving the neurobiological basis of apathy and impulsivity in neurodegenerative disease would facilitate the development and assessment of effective treatments and neuroprotective strategies. Herein, we focus on the heterogeneous clinical syndromes associated with FTLD, including behavioral variant frontotemporal dementia (bvFTD), primary progressive aphasias (nonfluent agrammatic variant [nvPPA], semantic variant [svPPA], and logopenic variant PPA), progressive supranuclear palsy (PSP), and the corticobasal syndrome (CBS).

We tested the hypothesis that across these diverse clinical syndromes, regionally specific pathology of white matter tracts as measured by diffusion tensor imaging (DTI)^7,8^ leads to different profiles of apathetic and impulsive behaviors.^9,10^ We consider the spectrum of FTLD disorders, rather than each separate syndrome, for 2 reasons. First, there is phenotypic overlap between syndromes.^3,11^ Second, apathy and impulsivity occur to a variable degree in each disorder,^3^ even where they are not diagnostic criteria. We predicted that separate dimensions of apathy and impulsivity would be associated with degeneration of distinct white matter tracts in neural systems supporting motivational and cognitive control.

## Methods

### Standard protocol approvals, registrations, and patient consents

The study was approved by the Cambridge 2 research ethics committee (reference 12/EE/0475) and supported by the National Institute for Health Research clinical research network (ID-15504). Informed consent was obtained at each study visit, with the personal consultee process used for participants who lacked mental capacity, in accordance with UK law.

### Participants

The Pick's Disease and Progressive Supranuclear Palsy: Prevalence and Incidence (PiPPIN) study recruited 204 participants. Recruitment and diagnostic criteria have been published previously.^11^ In brief, patients met clinical diagnostic criteria for behavioral^12^ and language^13^ variants of frontotemporal dementia (svPPA, nvPPA, logopenic variant PPA, and “other PPA” [not meeting criteria for 1 of the 3 defined subtypes]), CBS,^14^ and possible, probable, or definite PSP^15^ (predominantly PSP Richardson syndrome under the revised criteria^16^). Fifty healthy age- and sex-matched controls with no significant neurologic or psychiatric history were recruited. Participants were tested on their usual medication: 40% took “antidepressant” medications (for affective or behavioral indications), 29% dopaminergic medication, 4% antipsychotic medication, and 37% other centrally acting medications (benzodiazepines, antiepileptic, analgesics, pregabalin, or cholinesterase inhibitor). One hundred forty-nine patients and 50 controls underwent neuropsychological assessment, while advanced disease or death prevented assessment of the remaining patients.

One hundred participants underwent diffusion-weighted MRI. After quality control (excluding 1 patient and 2 controls), our imaging subset comprised 69 patients (22 PSP, 14 bvFTD, 14 CBS, 11 nvPPA, 4 svPPA, 4 other PPA) and 28 controls. To approximate group sizes, we evaluated PPA cases as a group. The scanned patients did not differ significantly from the nonscanned patients (table e-1, links.lww.com/WNL/A261).

### Cognitive and behavioral assessments

The test battery examined the major components of apathy and impulsivity (table 1). Questionnaires sought multiple perspectives, including clinician, patient, and carer. Computerized behavioral tasks included measures of response inhibition (restraint: Go/NoGo, and cancellation: stop signal task), reflection impulsivity (information sampling task), and reward sensitivity (cued reinforcement reaction time task, Cambridge Gambling Task). Saccade and motor versions of the Go/NoGo task were used in view of the motor impairment inherent to some FTLD syndromes. We also assessed potential confounds including depression (Beck Depression Inventory–II), anhedonia (Snaith-Hamilton Pleasure Scale), and akinesia (PSP Rating Scale and reaction times), as discussed in Lansdall et al.^3^

**Table 1:**
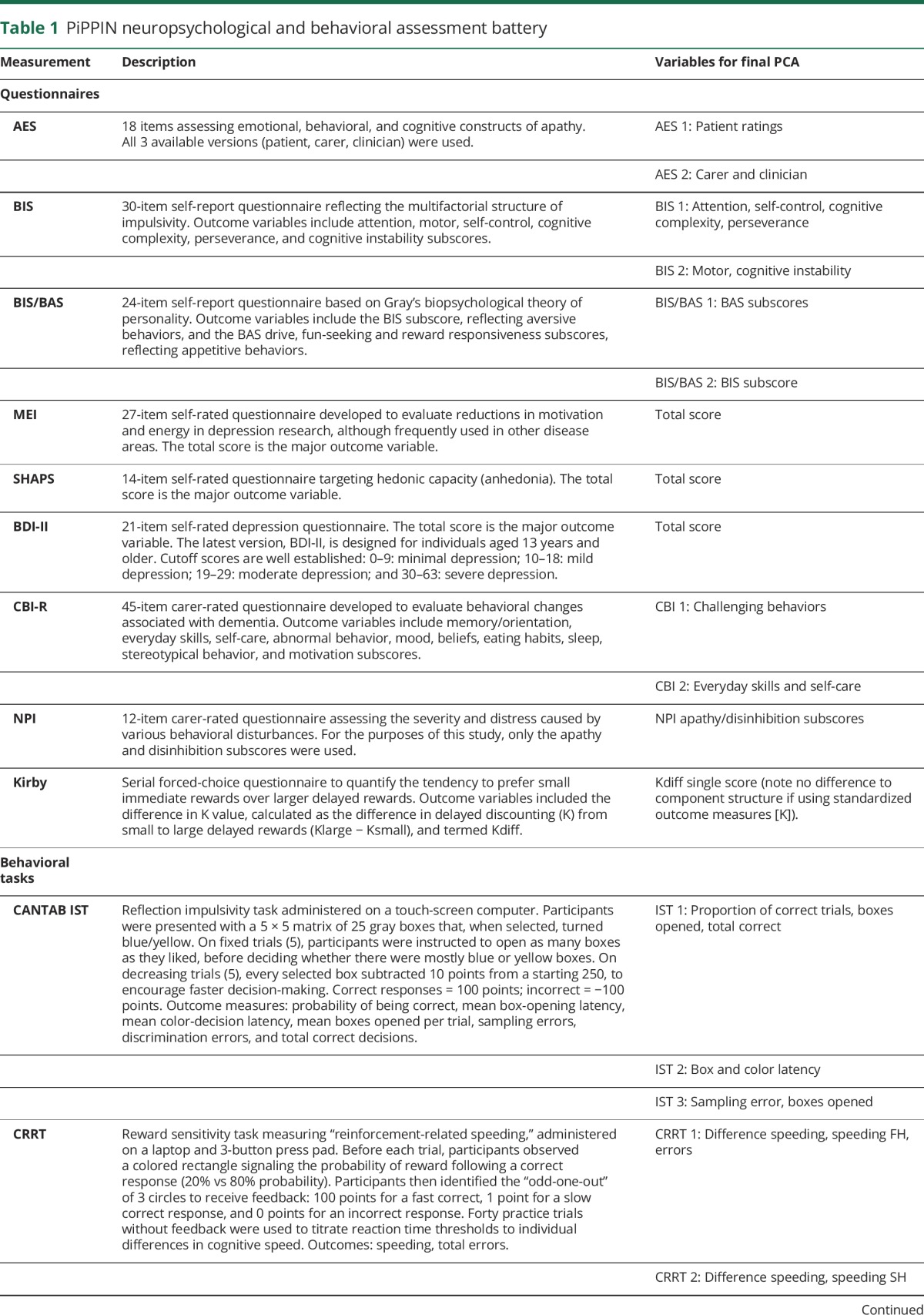

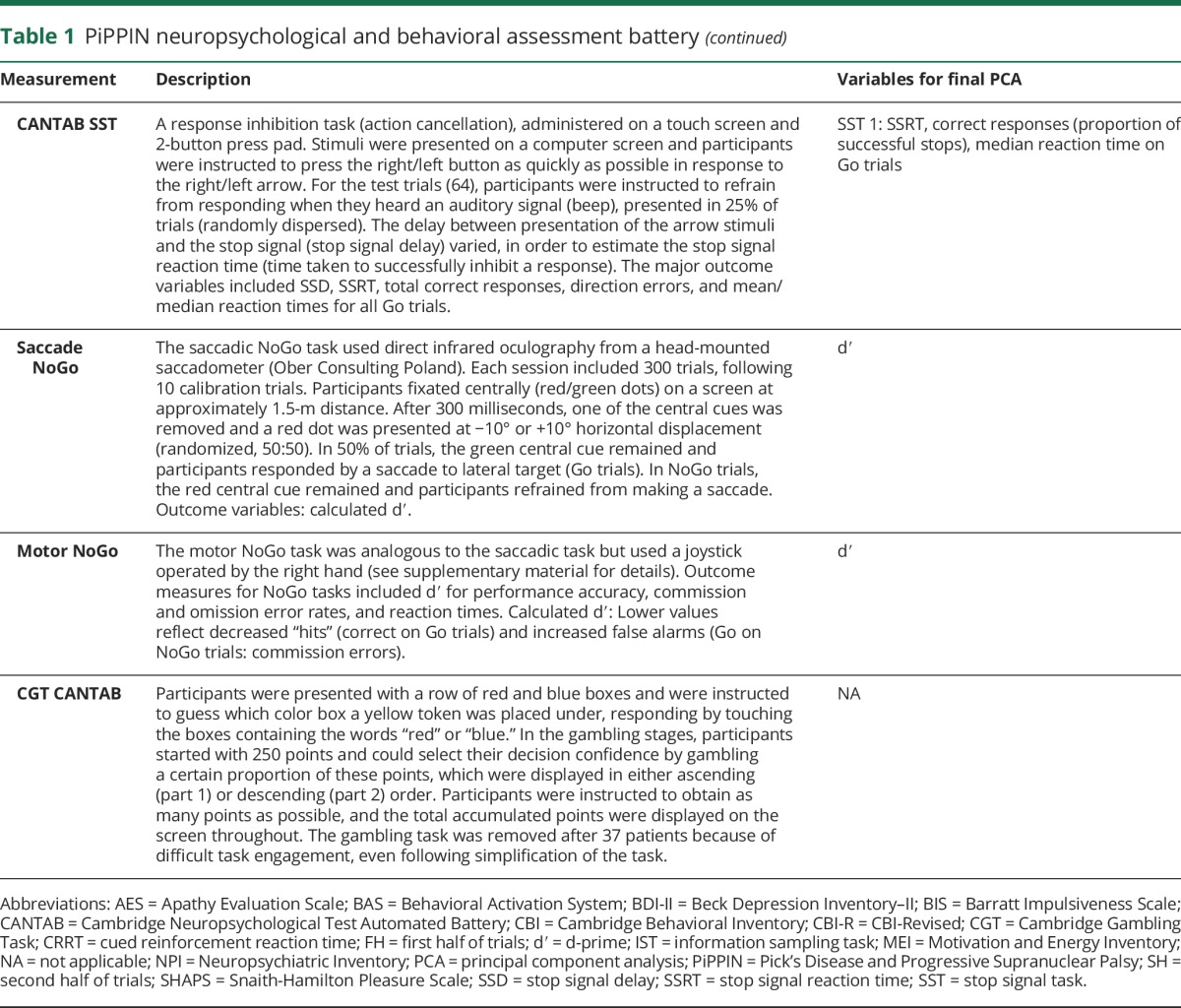
PiPPIN neuropsychological and behavioral assessment battery

SPSS version 22.0 (IBM Corp., Armonk, NY) was used for behavioral and neuropsychological analysis. Two-sample *t* tests, corrected for multiple comparisons, were used for group comparisons. Principal component analysis (PCA) identified the major components of apathy and impulsivity.^3^ In brief, PCAs were run on control and patient data combined (n = 199; noting no major difference to the component structure using patient data only) with varimax rotation and mean replacement for missing data. The correlation matrix was used for component extraction based on Kaiser and Cattell criteria (whichever was more inclusive), while Kaiser-Meyer-Olkin and Bartlett test of sphericity confirmed the adequacy of the sample for PCA. Where questionnaires or tasks had multiple outcome measures, we first ran a “local PCA.” A “final PCA” included the lead component loadings from local PCAs and total scores, accuracy (d-prime [d′]), or relevant subscores. Component scores were compared across groups using analysis of variance with post hoc least significant difference correction, and correlated with disease severity measures using Pearson correlations.

### Magnetic resonance imaging

Diffusion-weighted images were acquired using a Siemens Magnetom Tim Trio (Siemens, Erlangen, Germany) with a 63-direction gradient sequence with: b value 1,000 s/mm^2^; repetition time 7,800 milliseconds; echo time 90 milliseconds; axial in-plane acquisition matrix 96 × 96; field of view 192 × 192 mm; slice thickness 2 mm; and a total of 63 contiguous slices with in-plane resolution 2-mm isotropic. An additional b value of 0 s/mm^2^ image was acquired.

Images were processed using FMRIB Software Library (FSL version 5.0; www.fmrib.ox.ac.uk/fsl), correcting for eddy currents and participant motion by affine registration to the first b0 image (FSL eddy_correct); bvecs were rotated (fdt_rotate_bvecs). The b0 image was extracted and a brain mask created (Brain Extraction Tool). Diffusion tensors were fitted (dtifit) to create maps of fractional anisotropy (FA) and mean diffusivity (MD). FA maps from 5 participants from each group were nonlinearly registered to the FMRIB58_FA_1 mm target (tbss_2_reg). The warped FA images were averaged to produce a study-specific FA template.^17^ Registration was repeated for all participants using this study-specific FA template as target, bringing all participants into the same anatomical space. From the study-specific template, a mean FA skeleton was produced, and individual FA skeletons were mapped to it (threshold = 0.2). The transformations putting the individual FA maps into the skeletonized standard space were applied to MD maps.

Tract-based spatial statistics were used to examine the relationships between changes in diffusion metrics and behavior.^18^ Correlations between the skeleton DTI tracts and components of apathy and impulsivity were assessed by nonparametric permutation analysis using FSL randomise with threshold-free cluster enhancement (TFCE) correction, 2-dimensional optimization, and 5,000 permutations. The design matrix contained a constant term to model the intercept and each of the 8 orthogonal principal components of behavior. Cluster significance was tested at *p* < 0.01 and *p* < 0.05, corrected for multiple comparisons. White matter was labeled using the JHU (Johns Hopkins University) white-matter tractography atlas and ICBM-DTI-81 (International Consortium of Brain Mapping) white-matter labels atlas.

## Results

### Neuropsychological and behavioral results

Demographic, cognitive, neuropsychological, and behavioral results of patients and control participants who underwent DTI are displayed in table 2. Groups were matched for age and sex, while patients were impaired in cognition, disease severity, and most measures of apathy and impulsivity.

**Table 2:**
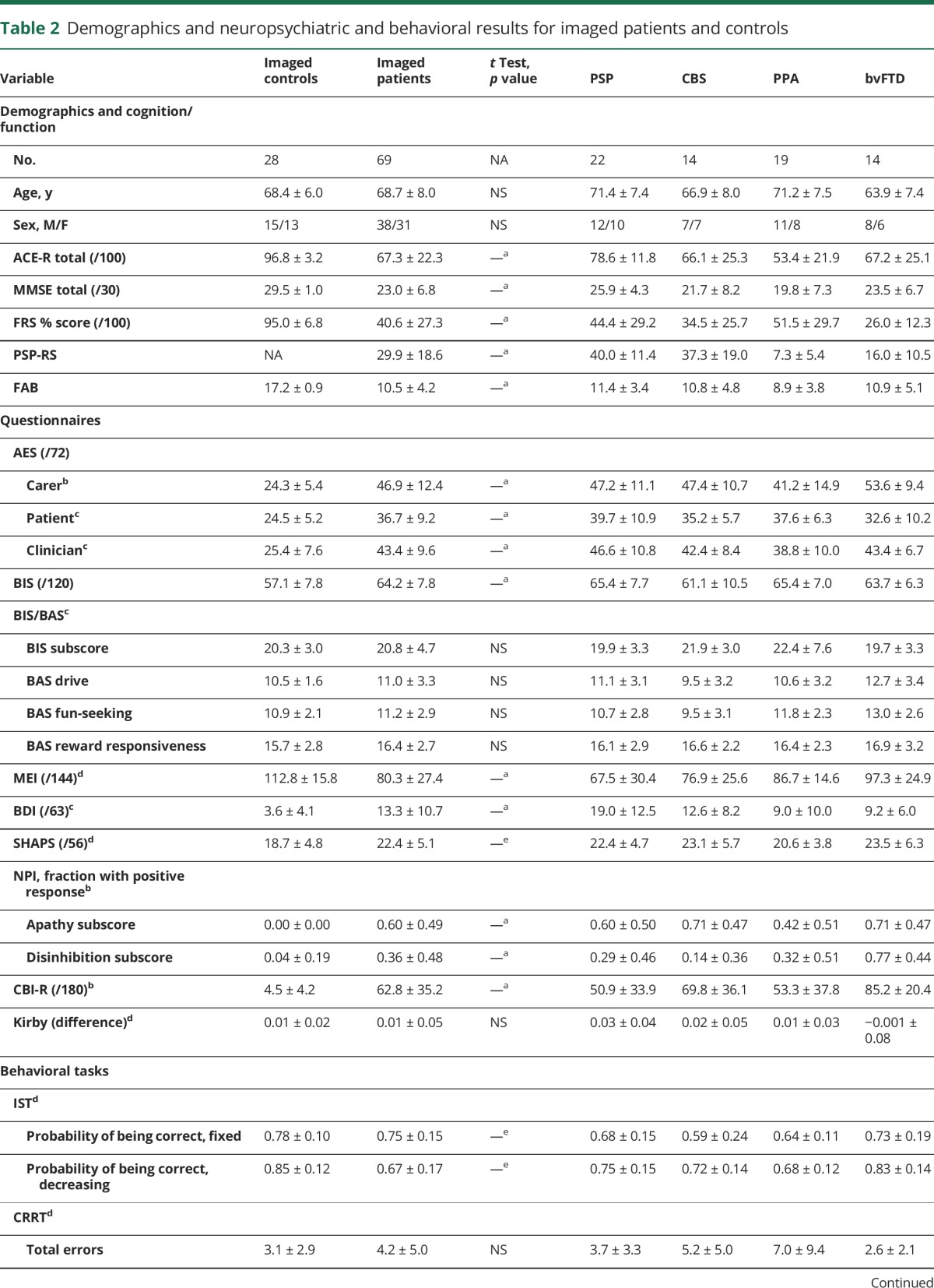

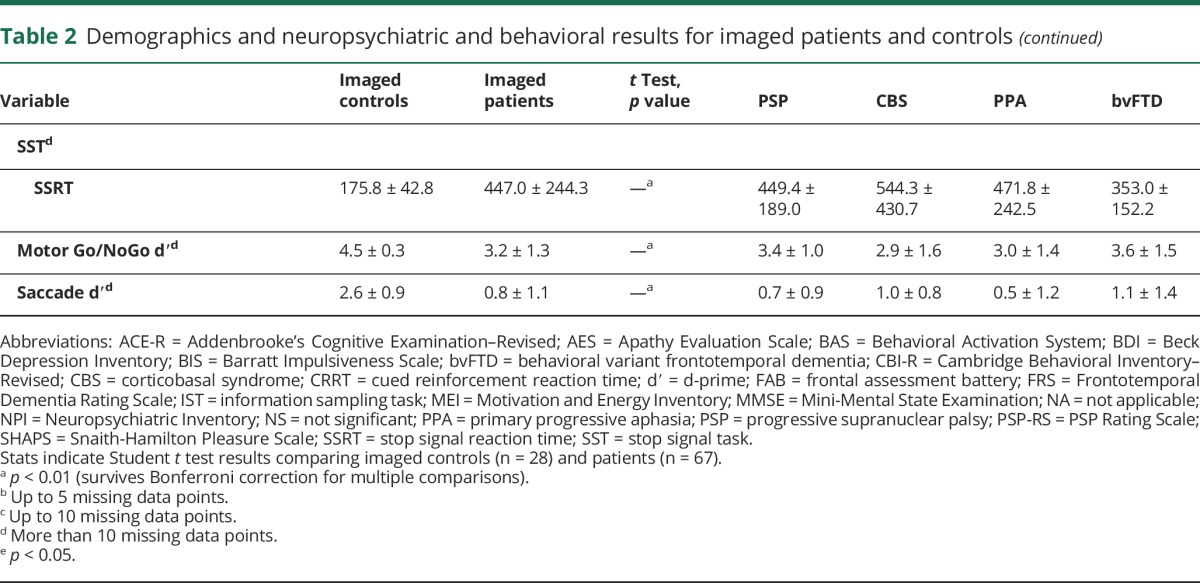
Demographics and neuropsychiatric and behavioral results for imaged patients and controls

The PCA identified 8 components (table 3; table e-2, links.lww.com/WNL/A261).^3^ Short summary terms were assigned to each according to their major loadings, after Lansdall et al.^3^ Component 1, termed “patient-rated change,” reflected self-ratings of apathy (Apathy Evaluation Scale [AES]), impulsivity (Barratt Impulsiveness Scale), anhedonia (Snaith-Hamilton Pleasure Scale), depression (Beck Depression Inventory–II), and motivation (Motivation and Energy Inventory). Components 2 and 3 were carer-based, weighted toward the AES, Cambridge Behavioral Inventory (CBI), and Neuropsychiatric Inventory (NPI); component 2, “carer-rated change in everyday skills/self-care,” reflected apathy (NPI apathy and AES), everyday skills, self-care, sleep, and motivation (CBI), while component 3, “carer-rated change in complex behaviors,” reflected apathy (AES), impulsivity (NPI disinhibition), and stereotypic/complex behaviors (CBI). Performance on the Go/NoGo, information sampling, and cued reinforcement tasks loaded onto component 4, termed “impulsive behavior.” Kaiser-Meyer-Olkin statistic = 0.743 and Bartlett test_231_ = 508; *p* < 0.001 confirmed data suitability for PCA. Patient-rated questionnaires, carer-rated questionnaires, and objective behavioral measures loaded onto distinct components, including positive weighting of both apathy and impulsivity measures.

**Table 3:**
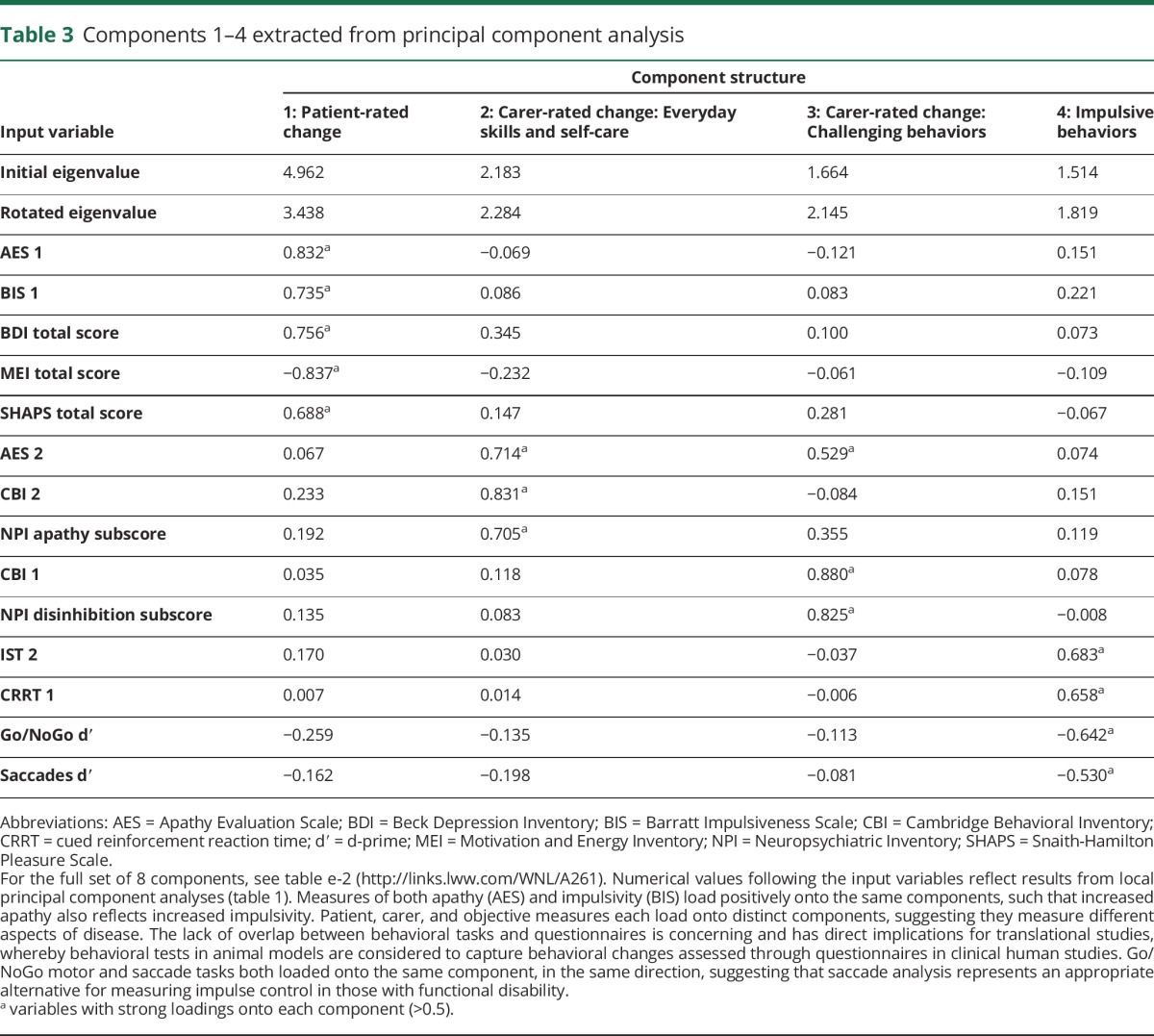
Components 1–4 extracted from principal component analysis

**Figure 1:**
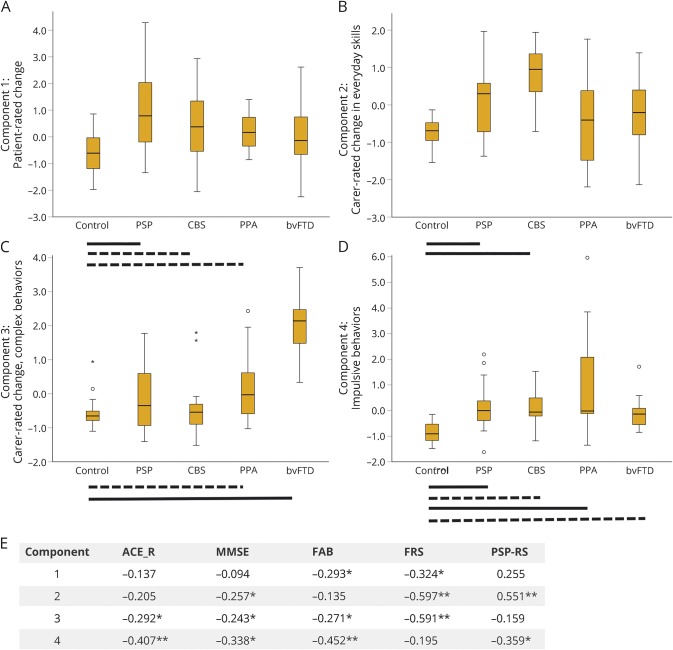
Component scores by diagnostic group (A–D) Boxplots of principal component scores (2–4) by diagnosis for the imaged subset (n = 97). Bars indicate significant differences between each group and controls using analysis of variance with post hoc least significant difference tests (solid lines *p* < 0.001, dashed lines *p* < 0.05), and circles/stars represent outliers (1.5*IQR/3*IQR, respectively). (A) Component 1 representing patient-rated behavioral change as measured by the AES, Barratt Impulsiveness Scale, Snaith-Hamilton Pleasure Scale, Beck Depression Inventory–II, and Motivation and Energy Inventory. (B) Component 2 reflecting carer-rated change in everyday skills, self-care, and motivation as measured by the CBI subscores, AES, and NPI apathy subscore. (C) Component 3 reflecting carer-rated change in complex behaviors as measured by the CBI abnormal/stereotypic behaviors, eating habits, mood and motivation subscores, AES, and NPI disinhibition subscore. (D) Component 4 indicating poor performance on behavioral tasks of response inhibition (Go/NoGo motor and saccade), reflection impulsivity (information sampling task), and reward responsiveness (cured reinforcement reaction time task). Significant differences were also observed between groups for component 1 (*F*_4,92_ = 7.462, *p* < 0.001, post hoc control vs PSP *p* < 0.001, vs CBS *p* < 0.05, vs PPA *p* < 0.05, PSP vs PPA *p* < 0.05, vs bvFTD *p* < 0.05), component 2 (*F*_4,92_ = 9.132, *p* < 0.001, post hoc control vs PSP *p* < 0.001, vs CBS *p* < 0.001, PSP vs CBS *p* < 0.05, vs PPA, *p* < 0.05, CBS vs PPA *p* < 0.001, vs bvFTD *p* = 0.001), component 3 (*F*_4,92_ = 23.832, *p* < 0.001, post hoc control vs bvFTD *p* < 0.001, vs PPA *p* < 0.05, PSP vs bvFTD *p* < 0.001, CBS vs bvFTD *p* < 0.001, PPA vs bvFTD *p* < 0.001), component 4 (*F*_4,92_ = 10.902, *p* < 0.001, post hoc control vs PSP *p* = 0.001, CBS *p* < 0.05, PPA *p* < 0.001, bvFTD *p* < 0.05, PSP vs PPA *p* < 0.05, CBS vs PPA *p* < 0.05, PPA vs bvFTD *p* = 0.001). (E) Components 1–4 correlated with measures of cognition (ACE-R, MMSE, FAB) and disease severity (FRS, PSP-RS) with higher component scores reflecting greater cognitive impairment, functional decline, and disease severity (note Pearson correlation, *p* < 0.001 uncorrected here approximates *p* < 0.05 corrected for multiple comparisons). * = *p* < 0.05; ** = *p* < 0.001 unc; ACE-R = Addenbrooke's Cognitive Examination–Revised; AES = Apathy Evaluation Scale; bvFTD = behavioral variant frontotemporal dementia; CBI = Cambridge Behavioral Inventory; CBS = corticobasal syndrome; FAB = frontal assessment battery; FRS = Frontotemporal Dementia Rating Scale; IQR = interquartile range; MMSE = Mini-Mental State Examination; NPI = Neuropsychiatric Inventory; PPA = primary progressive aphasia (all groups); PSP = progressive supranuclear palsy; PSP-RS = PSP Rating Scale.

Apathy and impulsivity were observed across the spectrum of clinical syndromes, reflecting their transdiagnostic nature. Significant differences between diagnostic groups were observed for loadings on components 1–4 (figure 1).

### Diffusion tensor imaging

**Figure 2:**
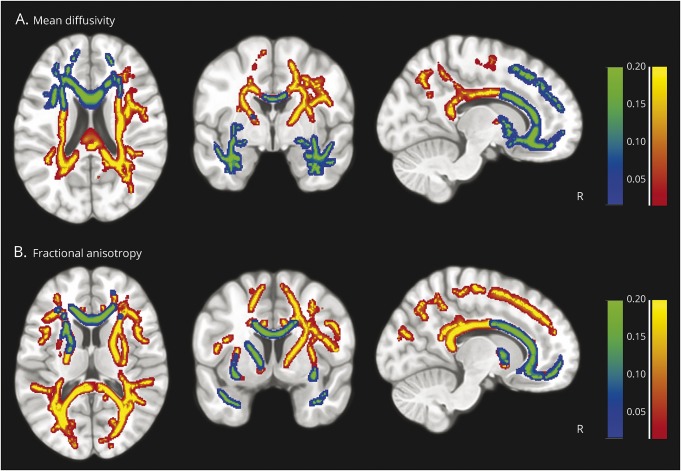
White matter changes associated with carer-rated everyday skills (component 2) and carer-rated complex behaviors (component 3) White matter correlates of carer-rated change in everyday skills and self-care (component 2: yellow-red) and carer-rated change in complex behaviors (component 3: blue-green), as measured by tract-based spatial statistics of diffusion tensor imaging. Correlations between the skeletonized diffusion tensor imaging–based tracts and the components were assessed by nonparametric permutation analysis using FSL randomise with threshold-free cluster enhancement correction, 2-dimensional optimization, and 5,000 permutations. Cluster significance was tested at *p* < 0.01 and corrected for multiple comparisons.

Tract-based spatial statistics identified significant (TFCE-corrected *p* < 0.01) changes in white matter in relation to carer-rated change in everyday skills and self-care (component 2, yellow-red) and carer-rated change in complex behaviors (component 3, blue-green; *p* < 0.01) (figure 2). Changes in MD and FA were complementary and highlighted concordant patterns of white matter change in relation to carer-rated change in everyday skills and self-care (component 2) and carer-rated change in complex behaviors (component 3). Loss of everyday skills correlated with FA (negative) and MD (positive) in the genu, body, and splenium of the corpus callosum, anterior and posterior corona radiata, corticospinal tracts, and posterior thalamic radiation (table e-3, links.lww.com/WNL/A261; figures e-1–e-3, links.lww.com/WNL/A260). Complex behaviors, including impulsivity, correlated with FA (negative) and MD (positive) in frontotemporal connections between the orbital- and ventrolateral-prefrontal cortex, anterior cingulate, and temporal pole, including the genu and body of the corpus callosum, anterior limb of the internal capsule, anterior thalamic radiation, and anterior corona radiata (figures e-4 and e-5). The anterior–posterior dissociation between components 3 and 2 is most apparent for MD (figure 2A). The longitudinal, fronto-occipital and uncinate fasciculi, and the forceps major and minor were associated with both carer-rated components, with a more restricted (anterior) distribution in relation to complex behaviors (figures 2, e-4, and e-5). At the more liberal threshold of *p* < 0.05 (TFCE-corrected), component 4 correlated with MD changes in regions connecting the presupplementary motor area and dorsolateral prefrontal cortex, and occipital lobe (thalamic radiation, forceps major, inferior fronto-occipital fasciculus, and inferior longitudinal fasciculus; figures e-1 and e-6).

## Discussion

Distinct spatial distributions of white matter pathology are related to separate dimensions of apathy and impulsivity, across multiple syndromes associated with FTLD. Carers' ratings of complex and challenging behaviors (including apathy and impulsivity, component 3) were associated with anterior changes in the white matter tracts connecting ventrolateral and orbitofrontal cortex and temporal poles. In contrast, carers' ratings of everyday skills, self-care, and apathy correlated with changes in frontal, parietal, and corticospinal tracts. Our data also show that (1) apathy and impulsivity are *positively* correlated, and (2) they are present in all syndromes associated with FTLD. These critical results reinforce the phenotypic overlap between disorders, reflected in new diagnostic terms such as PSP-CBS, PSP-F (frontal), and PSP-SL (speech/language).^16^ It also highlights the advantages of a dimensional approach that accommodates commonalties across groups and the convergence of syndromes with disease progression. In doing so, we confirm that even the language variants, especially svPPA, cause significant behavioral change including apathy and impulsivity.^2,3^

The white matter abnormalities associated with challenging behaviors (component 3: AES, NPI disinhibition, and CBI abnormal/stereotypic behaviors, eating habits, and motivation) are consistent with previous studies linking apathy and impulsivity to abnormal white matter and metabolism in frontotemporal regions.^1,2,4^ They mirror white matter tract abnormalities in bvFTD,^7,19^ for which apathy and impulsivity are diagnostic criteria. Moreover, the uncinate fasciculus is linked to inhibitory control in bvFTD and apathy in Alzheimer disease,^17^ small vessel disease,^20^ PSP,^21^ and bvFTD.^1^ This suggests a common neural pathway, across disorders.

Carer-rated change in everyday skills, self-care, motivation, and apathy correlated with widespread white matter changes in the corpus callosum, corona radiata, superior longitudinal fasciculus, and thalamic radiation. In contrast to carer-rated change in complex behaviors, there was less emphasis on rostral frontotemporal change. PSP and CBS groups scored most highly on this component, although all groups scored higher on average than controls (figure 1). The results support previous volumetric analyses showing the following: (1) PSP degeneration of the brainstem and association and commissural fibers including superior cerebellar peduncles, corpus callosum, inferior longitudinal fasciculus, and superior longitudinal fasciculus^21^; (2) CBS changes in frontoparietal tracts and corpus callosum^22^; and (3) FTD widespread changes.^23^

The widespread abnormalities are consistent with network-based disruption in FTLD,^24–26^ affecting broadly distributed frontotemporal networks rather than focal areas of damage. However, multifocal changes associated with carer reports may also reflect an inability to differentiate behavioral profiles using these questionnaires. Nonetheless, the tract-based statistics were broadly consistent with volumetric^3^ evidence of the breakdown of frontostriatal and frontotemporal circuits for motivation,^3,5^ coordinating the multiple cognitive domains necessary for planning and executing effective goal-directed behavior.^27^

One difference between the former volumetric study and current DTI results is the absence of a tract-based deficit in relation to patients' observations of their own symptomatology (table 3).^3^ There are several explanations for this discordance. First, patient ratings may reflect heterogeneous, multifocal changes in white matter, which prevent the identification of consistently localized tract correlates. Second, volumetric and DTI analyses assess fundamentally distinct neuropathologic features (tissue loss and T1 signal change vs the diffusional integrity of white matter connections), leading to different statistical associations. For example, patient ratings may reflect volumetric changes in deep white matter structures that are not captured by DTI. Third, the difference may reflect the limitations of white matter voxel-based morphometry,^18^ arising from normalization errors, mislocalization, or the partial-volume effects of smoothing, which can give rise to false-positives. The current tract-based method is less vulnerable to these issues, although there are limitations to the interpretation of DTI, which are discussed below. With the tract-based method, current white matter changes appear more extensive than the previously reported gray matter atrophy. For example, performance on the objective behavioral tasks correlated with white matter tract measures in the right frontal cortex, as well as white matter tracts near the regions of posterior and subcortical atrophy.^3^ This difference may be attributable to differential signal-to-noise of the 2 methods but may also reflect the core white matter pathophysiology in syndromes associated with FTLD.^7^

Although the behavioral task performance showed weaker correlations with MD and the carer ratings, its anatomical correlates are of particular relevance. First, all patient groups performed worse than controls (figure 1D), confirming the objective neuropsychological deficits as a transdiagnostic phenomenon. Second, these regions (presupplementary motor area, dorsolateral prefrontal cortex, and inferior frontal gyrus; figure e-1, links.lww.com/WNL/A260) and their interconnections are strongly associated with cognitive and motor control in preclinical models and human studies.^28–30^ Reduced connectivity among these regions affects response inhibition^31^ and choices between alternate actions.^32,33^

Carer ratings and behavioral task performance all correlated with cognitive and functional decline. Previous studies have reported a link between apathy and poor outcome,^20^ with rapid cognitive and functional deterioration in apathetic patients compared to nonapathetic and depressed individuals.^34^ Further investigations assessing the prognostic implications of apathy and impulsivity in FTLD syndromes are warranted.

There are limitations to this study and caveats to the methods. DTI is an indirect measure of the physical properties of brain parenchyma, including white matter axon density, caliber, and myelination.^35^ The pathologic causes of abnormal diffusion are not fully elucidated. Even though the semiquantitative in vivo measures provide important anatomical insights, cross-validation with neuropathology is sparse. For example, preclinical studies link FA to myelination, membrane permeability, and fiber density in white matter.^36^ Comparative studies of anatomy across species and in FTLD post mortem are required to determine the pathologic mechanisms of the imaging changes we observe. Although different DTI metrics may reflect distinct processes (demyelination, neurodegeneration, gliosis, calcification, axonal degeneration, etc.), linking them to specific leucopathologies remains challenging. One must also consider artifacts from motion and registration errors, as multiple directional measurements are obtained at each voxel, introducing false-positive differences if movement differs by group.^37^ Registration poses significant challenges for FTLD groups with highly atrophic brains, obscuring some tracts and affecting the absolute diffusivities or eigenvalues.^38^ White matter change in areas with substantial gray matter atrophy may lead to changes in estimated FA/MD that reflect differences in the relative amounts of tissue types rather than change in white matter.^18^ In addition to these general DTI considerations, tract-based spatial statistics also has caveats. It attempts to overcome misregistration issues and ensure the same region (or voxel) corresponds across groups by creating a mean FA skeleton, onto which each individual's FA is projected prior to statistics. This relies on accurate coregistration of FA images. White matter lesions that reduce FA may also alter the values chosen to represent the core of the tract during FA projection.^39^ Despite these risks, we favor tract-based spatial statistics over other frequently used whole-brain methods because of its increased sensitivity^40^ and power.^39^

A neuropsychological battery is necessarily selective, and our findings are limited to the patients studied and the dimensions of apathy and impulsivity accessible to our tests and questionnaires. PiPPIN aimed to assess the multifaceted constructs of apathy and impulsivity, while accommodating the frailty of patients. Nonetheless, many patients found the Cambridge Gambling Task difficult to perform adequately. However, pathologic gambling is uncommon in FTLD disorders, and including this task in a subsidiary PCA did not alter the factor structure. Alternative tasks and questionnaires (e.g., cued reinforcement reaction time) remained, to assess motivation and reward. We acknowledge that questionnaires are limited in their ability to determine the underlying cause of behavioral change. For example, answering “he/she shows less enthusiasm for his or her usual interests” may be confounded by learned restrictions arising from physical motor impairments, or be influenced by semantic impairments and executive deficits. By using many tasks across a number of populations, we suggest that the extracted dimensions of apathy and impulsivity more accurately capture the essence of these behavioral changes than the use of single questions or tasks in isolation.

Finally, although PiPPIN aimed to be representative of the full population of affected patients, some may not have a diagnosis or be in contact with referring services. We also rely on clinical diagnostic criteria and acknowledge that some variants (svPPA, PSP) have much stronger clinic–pathologic correlations than others (CBS, bvFTD). Although we used multiple sources of referral in community and specialist services to reach all patients within the catchment area, some may have been missed. Nonetheless, the imaging subset was similar to the whole cohort.

White matter is markedly abnormal in the clinical syndromes associated with FTLD. DTI was sensitive to the white matter changes underlying FTLD-associated behaviors and revealed distinct spatial profiles relating to different aspects of apathy and impulsivity. These complex, multifaceted constructs are common across the FTLD spectrum and remain poorly treated. Elucidating the neural correlates of apathy and impulsivity, transdiagnostically, will help to inform the design of clinical trials for novel therapeutic strategies.

## Glossary

AES: Apathy Evaluation Scale
bvFTD: behavioral variant frontotemporal dementia
CBI: Cambridge Behavioral Inventory
CBS: corticobasal syndrome
DTI: diffusion tensor imaging
FA: fractional anisotropy
FTD: frontotemporal dementia
FTLD: frontotemporal lobar degeneration
MD: mean diffusivity
NPI: Neuropsychiatric Inventory
nvPPA: nonfluent agrammatic variant primary progressive aphasia
PCA: principal component analysis
PiPPIN: Pick's Disease and Progressive Supranuclear Palsy: Prevalence and Incidence
PPA: primary progressive aphasia
PSP: progressive supranuclear palsy
svPPA: semantic variant primary progressive aphasia
TFCE: threshold-free cluster enhancement

## References

[1] Powers J, Massimo L, McMillan C, et al. White matter disease contributes to apathy and disinhibition in behavioural variant frontotemporal dementia. Cogn Behav Neurol 2014;27:206–214. 25539040 10.1097/WNN.0000000000000044 PMC4296883

[2] Zamboni G, Huey ED, Krueger F, et al. Apathy and disinhibition in frontotemporal dementia: insights into their neural correlates. Neurology 2008;71:736–742. 18765649 10.1212/01.wnl.0000324920.96835.95 PMC2676948

[3] Lansdall CJ, Coyle-Gilchrist I, Jones P, et al. Apathy and impulsivity in frontotemporal lobar degeneration syndromes. Brain 2017;140:1792–1807. 28486594 10.1093/brain/awx101 PMC5868210

[4] Massimo L, Powers C, Moore P, et al. Neuroanatomy of apathy and disinhibition in frontotemporal lobar degeneration. Dement Geriatr Cogn Disord 2009;27:96–104. 19158440 10.1159/000194658 PMC2820577

[5] Levy R, Dubois B. Apathy and the functional anatomy of the prefrontal cortex–basal ganglia circuits. Cereb Cortex 2006;16:916–928. 16207933 10.1093/cercor/bhj043

[6] Dalley JW, Everitt BJ, Robbins TW. Impulsivity, compulsivity and top-down cognitive control. Neuron 2011;69:680–694. 21338879 10.1016/j.neuron.2011.01.020

[7] Mahoney CJ, Ridgway GR, Malone IB, et al. Profiles of white matter tract pathology in frontotemporal dementia. Hum Brain Mapp 2014;35:4163–4179. 24510641 10.1002/hbm.22468 PMC4312919

[8] Ghosh BCP, Calder AJ, Peers PV, et al. Social cognitive deficits and their neural correlates in progressive supranuclear palsy. Brain 2012;135:2089–2102. 22637582 10.1093/brain/aws128 PMC3381722

[9] Burrell JR, Hodges JR, Rowe J. Cognition in corticobasal syndrome and progressive supranuclear palsy: a review. Mov Disord 2014;29:684–693. 24757116 10.1002/mds.25872

[10] Zhang J, Rittman T, Nombela C, et al. Different decision deficits impair response inhibition in progressive supranuclear palsy and Parkinson's disease. Brain 2016;139:161–173. 26582559 10.1093/brain/awv331 PMC4949391

[11] Coyle-Gilchrist ITS, Dick KM, Patterson K, et al. Prevalence, characteristics, and survival of frontotemporal lobar degeneration syndromes. Neurology 2016;86:1736–1743. 27037234 10.1212/WNL.0000000000002638 PMC4854589

[12] Rascovsky K, Hodges JR, Knopman D, et al. Sensitivity of revised diagnostic criteria for the behavioural variant of frontotemporal dementia. Brain 2011;134:2456–2477. 21810890 10.1093/brain/awr179 PMC3170532

[13] Gorno-Tempini ML, Hillis AE, Weintraub S, et al. Classification of primary progressive aphasia and its variants. Neurology 2011;76:1006–1014. 21325651 10.1212/WNL.0b013e31821103e6 PMC3059138

[14] Armstrong MJ, Litvan I, Lang AE, et al. Criteria for the diagnosis of corticobasal degeneration. Neurology 2013;80:496–503. 23359374 10.1212/WNL.0b013e31827f0fd1 PMC3590050

[15] Litvan I, Agid Y, Calne D, et al. Clinical research criteria for the diagnosis of progressive supranuclear palsy (Steele-Richardson-Olszewski syndrome): report of the NINDS-SPSP International Workshop. Neurology 1996;47:1–9. 8710059 10.1212/wnl.47.1.1

[16] Höglinger G, Respondek G, Stamelou M, et al; Movement Disorder Society–endorsed PSP Study Group. Clinical diagnosis of progressive supranuclear palsy: the Movement Disorder Society criteria. Mov Disord 2017;32:853–864. 28467028 10.1002/mds.26987 PMC5516529

[17] Douaud G, Jbabdi S, Behrens TEJ, et al. DTI measures in crossing-fibre areas: increased diffusion anisotropy reveals early white matter alteration in MCI and mild Alzheimer's disease. Neuroimage 2011;55:880–890. 21182970 10.1016/j.neuroimage.2010.12.008 PMC7116583

[18] Smith SM, Jenkinson M, Johansen-Berg H, et al. Tract-based spatial statistics: voxelwise analysis of multi-subject diffusion data. Neuroimage 2006;31:1487–1505. 16624579 10.1016/j.neuroimage.2006.02.024

[19] Tovar-Moll F, De Oliveira-Souza R, Bramati IE, et al. White matter tract damage in the behavioral variant of frontotemporal and corticobasal dementia syndromes. PLoS One 2014;9:e102656. 25054218 10.1371/journal.pone.0102656 PMC4108323

[20] Hollocks MJ, Lawrence AJ, Brookes RL, et al. Differential relationships between apathy and depression with white matter microstructural changes and functional outcomes. Brain 2015;138:3803–3815. 26490330 10.1093/brain/awv304 PMC4655344

[21] Whitwell JL, Master AV, Avula R, et al. Clinical correlates of white matter tract degeneration in PSP. Arch Neurol 2011;68:753–760. 21670399 10.1001/archneurol.2011.107 PMC3401587

[22] Borroni B, Garibotto V, Agosti C, et al. White matter changes in corticobasal degeneration syndrome and correlation with limb apraxia. Arch Neurol 2008;65:796–801. 18541800 10.1001/archneur.65.6.796

[23] Agosta F, Galantucci S, Magnani G, et al. MRI signatures of the frontotemporal lobar degeneration continuum. Hum Brain Mapp 2015;36:2602–2614. 25821176 10.1002/hbm.22794 PMC6869605

[24] Seeley WW, Crawford R, Rascovsky K, et al. Frontal paralimbic network atrophy in very mild behavioural variant frontotemporal dementia. Arch Neurol 2008;65:249–255. 18268196 10.1001/archneurol.2007.38 PMC2544627

[25] Rae CL, Nombela C, Vázquez Rodriguez P, et al. Atomoxetine restores the response inhibition network in Parkinson's disease. Brain 2016;139:2235–2248. 27343257 10.1093/brain/aww138 PMC4958901

[26] Hughes LE, Ghosh BCP, Rowe JB. Reorganisation of brain networks in frontotemporal dementia and progressive supranuclear palsy. Neuroimage Clin 2013;2:459–468. 23853762 10.1016/j.nicl.2013.03.009 PMC3708296

[27] Haber SN. Corticostriatal circuitry. Dialogues Clin Neurosci 2016;18:7–21. 27069376 10.31887/DCNS.2016.18.1/shaber PMC4826773

[28] Ye Z, Altena E, Nombela C, et al. Improving response inhibition in Parkinson's disease with atomoxetine. Biol Psychiatry 2015;77:740–748. 24655598 10.1016/j.biopsych.2014.01.024 PMC4384955

[29] Aron AR, Fletcher PC, Bullmore ET, Sahakian BJ, Robbins TW. Stop-signal inhibition disrupted by damage to right inferior frontal gyrus in humans. Nat Neurosci 2003;6:115–116. 12536210 10.1038/nn1003

[30] Perry R, Miller B. Behavior and treatment in frontotemporal dementia. Neurology 2001;56:S46–S51. 11402151 10.1212/wnl.56.suppl_4.s46

[31] Forstmann BU, Anwander A, Schäfer A, et al. Cortico-striatal connections predict control over speed and accuracy in perceptual decision making. Proc Natl Acad Sci USA 2010;107:15916–15920. 20733082 10.1073/pnas.1004932107 PMC2936628

[32] Rushworth MF. Intention, choice, and the medial frontal cortex. Ann NY Acad Sci 2008;207:181–207. 10.1196/annals.1440.014 18400931

[33] Rowe JB, Hughes L, Nimmo-Smith I. Action selection: a race model for selected and non-selected actions distinguishes the contribution of premotor and prefrontal areas. Neuroimage 2010;51:888–896. 20188184 10.1016/j.neuroimage.2010.02.045 PMC2877799

[34] Vicini Chilovi B, Conti M, Zanetti M, et al. Differential impact of apathy and depression in the development of dementia in mild cognitive impairment patients. Dement Geriatr Cogn Disord 2009;27:390–398. 19339777 10.1159/000210045

[35] Jbabdi S, Sotiropoulos SN, Haber SN, Van Essen DC, Behrens TE. Measuring macroscopic brain connections in vivo. Nat Neurosci 2015;18:1546–1555. 26505566 10.1038/nn.4134

[36] Song S, Sun S, Ju W, Lin SJ, Cross AH, Neufeld AH. Diffusion tensor imaging detects and differentiates axon and myelin degeneration in mouse optic nerve after retinal ischemia. Neuroimage 2003;20:1714–1722. 14642481 10.1016/j.neuroimage.2003.07.005

[37] Pardoe HR, Hiess RK, Kuzniecky R. Motion and morphometry in clinical and nonclinical populations. Neuroimage 2016;135:177–185. 27153982 10.1016/j.neuroimage.2016.05.005

[38] Zhang Y, Carmela M, Schuff N, Chiang GC. MRI signatures of brain macrostructural atrophy and microstructural degradation in frontotemporal lobar degeneration subtypes. J Alzheimers Dis 2013;33:431–444. 22976075 10.3233/JAD-2012-121156 PMC3738303

[39] Jones DK, Cercignani M. Twenty-five pitfalls in the analysis of diffusion MRI data. NMR Biomed 2010;23:803–820. 20886566 10.1002/nbm.1543

[40] Rae CL, Correia MM, Altena E, Hughes LE, Barker RA, Rowe JB. White matter pathology in Parkinson's disease: the effect of imaging protocol differences and relevance to executive function. Neuroimage 2012;62:1675–1684. 22713671 10.1016/j.neuroimage.2012.06.012 PMC3413883

